# Referring physicians underestimate the extent of abnormalities in final reports from myocardial perfusion imaging

**DOI:** 10.1186/2191-219X-2-27

**Published:** 2012-06-09

**Authors:** Elin Trägårdh, Peter Höglund, Mattias Ohlsson, Mattias Wieloch, Lars Edenbrandt

**Affiliations:** 1 Clinical Physiology and Nuclear Medicine Unit, Skåne University Hospital, Lund University, Entrance 44, Malmö, 205 05, Sweden; 2 Competence Center for Clinical Research, Skåne University Hospital, Lund, Sweden; 3 Computational Biology and Biological Physics, Lund University, Lund, Sweden; 4 Department of Cardiology, Skåne University Hospital, Lund University, Malmö, Sweden

**Keywords:** Structured reporting, Ischemic heart disease, 99mTc MPS, Infarction, Ischemia

## Abstract

**Background:**

It is important that referring physicians and other treating clinicians properly understand the final reports from diagnostic tests. The aim of the study was to investigate whether referring physicians interpret a final report for a myocardial perfusion scintigraphy (MPS) test in the same way that the reading nuclear medicine physician intended.

**Methods:**

After viewing final reports containing only typical clinical verbiage and images, physicians in nuclear medicine and referring physicians (physicians in cardiology, internal medicine, and general practitioners) independently classified 60 MPS tests for the presence versus absence of ischemia/infarction according to objective grades of 1–5 (1 = No ischemia/infarction, 2 = Probably no ischemia/infarction 3 = Equivocal, 4 = Probable ischemia/infarction, and 5 = Certain ischemia/infarction). When ischemia and/or infarction were thought to be present in the left ventricle, all physicians were also asked to mark the involved segments based on the 17-segment model.

**Results:**

There was good diagnostic agreement between physicians in nuclear medicine and referring physicians when assessing the general presence versus absence of both ischemia and infarction (median squared kappa coefficient of 0.92 for both). However, when using the 17-segment model, compared to the physicians in nuclear medicine, 12 of 23 referring physicians underestimated the extent of ischemic area while 6 underestimated and 1 overestimated the extent of infarcted area.

**Conclusions:**

Whereas referring physicians gain a good understanding of the general presence versus absence of ischemia and infarction from MPS test reports, they often underestimate the extent of any ischemic or infarcted areas. This may have adverse clinical consequences and thus the language in final reports from MPS tests might be further improved and standardized.

## **Background**

Whenever diagnostic tests are performed, it is important that referring physicians fully understand the final report, written for example by other physicians who are most often radiologists or pathologists. If the message in the final report is not precisely understood, the patient might receive inadequate, inappropriate or potentially even harmful treatment. Numerous studies have investigated the sources for potential clinical errors in many different diagnostic methods, including technical aspects and inter- and intra observer variability. However, to our knowledge, none have investigated whether the referring physician understands the message sent in the final report by the diagnostic physician.

Stress myocardial perfusion scintigraphy (MPS) is widely regarded as a clinically useful non-invasive imaging modality for diagnosing patients with suspected coronary artery disease [1-3]. When a physician refers a patient to a MPS, the physician wants to know whether ischemia and/or infarction are present, as well as the extent and severity of any perfusion defects. In order to optimally manage the patient, the referring physician should fully understand the final report generated by the nuclear medicine specialist. The American Society of Nuclear Cardiology, European Association of Nuclear Medicine and European Society of Cardiology have published guidelines, articles and editorials that address the importance of reporting in an understandable manner [4-10].

Current guidelines recommend that MPS reports should be concise and couched in language that is easily understandable to referring physicians [10]. As a standard, the reports should consist of information about patient details, indication(s) for study, stress technique, tracer and imaging protocol, findings, and conclusion. In the conclusion section, information about left ventricular perfusion (presence/absence of inducible ischemia and/or infarction), left ventricular function (global and regional function, possible stress-induced abnormalities), inconclusive study (may occasionally be the correct conclusion) and correlation with and deviations from clinical information and other data if available, should be included.

The principal aim of the present study was to investigate whether referring physicians (cardiologists, internists and general practitioners) interpret the final reports from MPS tests in the same way as intended by the nuclear medicine physicians who generate the written final reports. A secondary aim was to examine the details of any differences in objective classifications that occur between referring and nuclear medicine physicians.

## **Methods**

**Figure 1:**
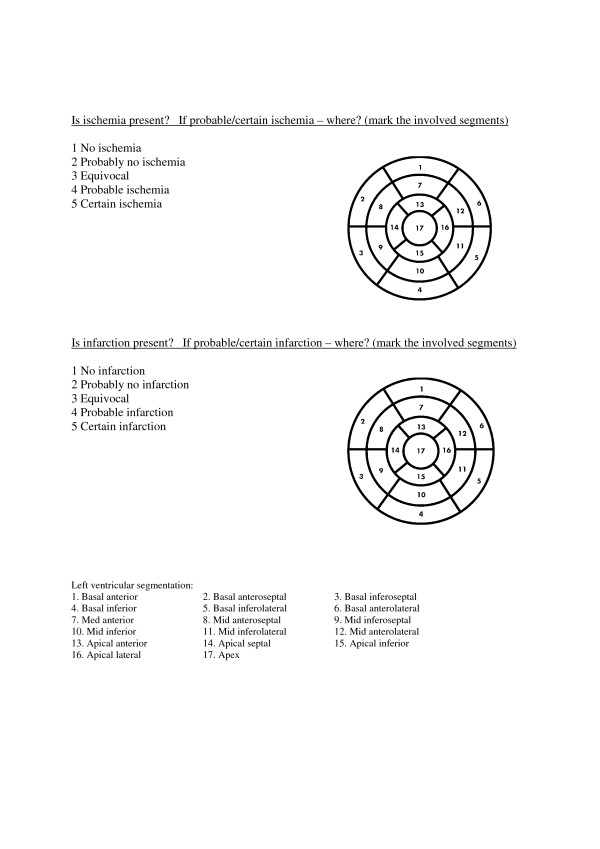
The questionnaire filled in by both the physicians at the nuclear medicine department and referring physicians.

Initially, MPS tests performed at the Department of Nuclear Medicine, Skåne University Hospital, Malmö, Sweden during January-July 2011 were considered for inclusion in the study. Tests performed by six of the physicians at the department were included (one resident with 1.5 years of experience in MPS, whose final reports were approved by a senior physician, and 5 specialists in nuclear medicine and/or clinical physiology). The MPS tests were selected by one study investigator (ET) in order to include 10 results from each of the six nuclear medicine physicians, of which only 3 tests from each physician had completely normal results. For results to be considered “completely normal”, both “no infarction” and “no inducible ischemia” had to be clearly indicated in the conclusion section of the final report, i.e. the study investigator who selected the cases was to be almost sure that the report should be assessed as certainly “no ischemia” and “no infarction”. Only 3 completely normal results from each physician were chosen in order to obtain a larger variety of reports with abnormalities because in our hospital’s laboratory, more than 50% of MPS test results are considered normal. Each of the physicians in nuclear medicine were asked to fill in a questionnaire (Figure 1) based on the final reports for their own 10 included patients. They then assessed the tests based on the presence versus absence of ischemia/infarction in grades of 1–5 (1 = No ischemia/infarction, 2 = Probably no ischemia/infarction 3 = Equivocal, 4 = Probable ischemia/infarction, and 5 = Certain ischemia/infarction). When ischemia and/or infarction were thought to be present in the left ventricle, the same physicians were then also asked to mark the involved segments based on the 17-segment model. The only other information provided beyond the original written final report and images for the MPS was patient age and gender.

Thirty physicians from specialties who often refer patients for MPS tests were presented with the 60 final reports, and were asked to fill in the same questionnaire. Of these, 10 were cardiologists, 10 were internists and 10 were general practitioners. Twenty-three physicians chose to participate in the study: 8 cardiologists, 7 internists, and 8 general practitioners. Twelve of these physicians were already specialists and 11 were residents.

### **MPS**

The MPS tests were performed per clinical routine in our department, using a 2-day gated stress/non-gated rest Tc-99 m-tetrofosmin protocol, starting with an injection of 600 MBq Tc-99 m-tetrofosmin at stress. Patients were stressed either by maximal exercise (ergometry) or pharmacologically by adenosine. The exercise test was continued for at least 1 min after the injection of the tracer and the adenosine infusion for at least 2 min after the injection of the tracer. Normal findings at stress were not followed by a rest study. Stress studies that were not completely normal were followed by a rest study with injection of 600 MBq Tc-99 m-tetrofosmin.

Stress and rest acquisition began about 60 min after the end of the injection of Tc-99 m-tetrofosmin. Images were obtained according to established clinical protocols, using single photon emission computed tomography over 180° elliptical, autocontour rotations from the 45° right anterior oblique position, with a dual-head gamma camera, e.cam (Siemens AG Medical Solutions, Erlangen, Germany). Patients were imaged in the supine position. A low energy high-resolution collimator and a zoom factor of 1.0 were used. We obtained 64 (32 views per camera) projections in a 128 x 128 matrix, with an acquisition time of 20 s per projection. Stress images were gated to the electrocardiogram using 8 frames per cardiac cycle. No automatic motion-correction program was applied; instead the acquisition was repeated if motion was detected. Tomographic reconstruction and calculation of short and long axis slice images were performed using e.soft (Siemens AG Medical Solutions, Erlangen, Germany). Non-attenuation corrected images were reconstructed with filtered back-projection. A 2D Butterworth pre-reconstruction filter was used with cut-off frequency of 0.45, order 5. Attenuation corrected images were reconstructed with an iterative algorithm, 6 iterations where a ramp filter was applied on the error projection prior to backprojection. A Butterworth filter with a cut-off frequency of 0.40, order 5, was applied for regularization. Attenuation maps were generated from simultaneous transmission measurement using a Gd-153 multiple-line source (Siemens AG Medical Solutions, Erlangen, Germany).

### **Reports**

**Figure 2:**
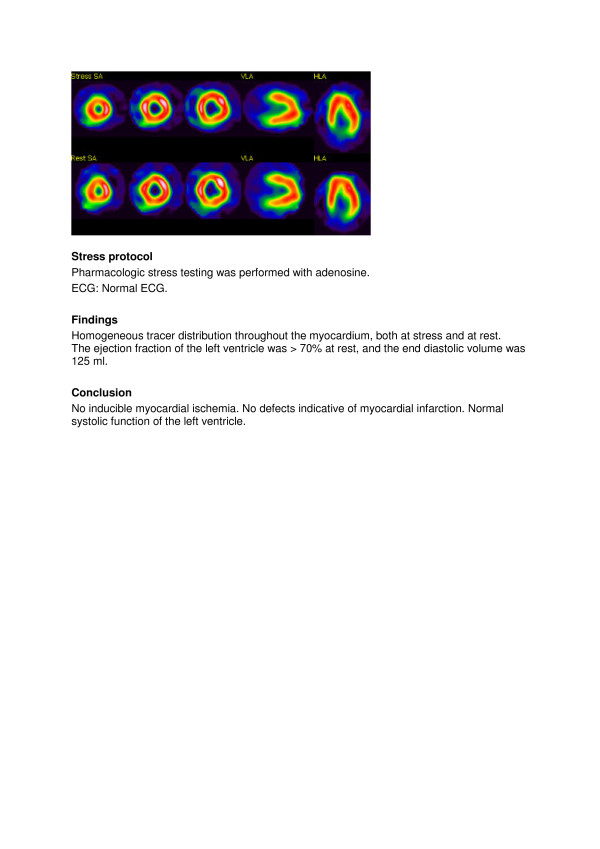
Example of a routine MPS test result reported as normal.

The reports were written according to local clinical routine in EXINI heart^TM^ (EXINI Diagnostics AB, Lund Sweden), and typically consisted of three headings: Stress technique, Findings, and Conclusion. Copies of the images, chosen by the interpreting physician, accompanied the report: three short axis slices (apical, mid, basal), one vertical long axis and one horizontal long axis for stress images. For rest images, the corresponding slices were shown, aligned to the stress images. Attenuation corrected images were used by default, but if the interpreting physician elected to include non-attenuation corrected images instead, this was possible. A typical normal report is shown in Figure 2.

### **Statistical analysis**

For analysis of agreement between the physicians in nuclear medicine and the referring physicians, the percentage agreement (PA) and the squared kappa coefficient (which measures agreement beyond that expected by chance) were calculated. Squared kappa was used because disagreement by an increasing number of grades on the five-grade scale potentially has increasingly more serious clinical consequences.

Disagreement between the physician in nuclear medicine and the referring physician can be systematic and/or random. To quantify the disagreement between paired-ordered categorical classifications, a method by Svensson et al [11,12] was used. Two types of systematic variation are possible: overestimation or underestimation of the classifications and concentration of the classification. Systematic overestimation or underestimation occurs when an observer regularly classifies cases as being more or less abnormal than another observer does. This is reflected by the variable relative position (RP) and possible values range from −1 to 1. A value of 0 indicates that no systematic disagreement is present. A positive RP value reflects systematic overestimation of the classifications, while a negative RP value reflects systematic underestimation.

Systematic concentration occurs when an observer more often uses the middle part of the five-grade scale (grades 2–4) than another observer who uses “no ischemia/infarction” or “certain ischemia/infarction” (grades 1 and 5) more often. This is reflected by the variable relative concentration (RC). The possible values for RC also range from −1 to 1, where a value of 0 indicates that no systematic disagreement is present. The RC value is positive if systematic concentration to the central part of the five-grade scale is present and negative when systematic concentration to the extremity grades is present.

The pattern of random differences was quantified using the variable of relative rank variance (RV). Random errors could be caused by guessing or loss of concentration. The possible values for RV range from 0 to 1, with 0 indicating no random contribution.

For the analysis of differences in the interpretation of the 17-segment model for presence/absence of ischemia/infarction, the Durkalski method was used [13]. This is a method for the analysis of clustered matched-pair data that adjusts for multiple units within a cluster, yet avoids correlation assumptions among and within clusters and also avoids distributional assumptions (modification of the McNemar test). The level of statistical significance was set at p < 0.05.

## **Results**

### **Assessment of ischemia and infarction made by the nuclear medicine physician**

In relation to ischemia, the nuclear medicine physicians classified the result as “no ischemia” in 32 cases, as “probably no ischemia” in 5 cases, as “equivocal” in 0 cases, as “probable ischemia” in 14 cases, and as “certain ischemia” in 9 cases. In relation to infarction, the nuclear medicine physicians classified the result as “no infarction” in 31 cases, as “probably no infarction” in 3 cases, as “equivocal” in 3 cases, as “probable infarction” in 7 cases and as “certain infarction” 16 cases. In total, 18 of the 60 tests were considered completely normal by the nuclear medicine physicians.

### **Analysis of classification of ischemia**

Table 1 shows squared kappa, PA, RC, RP and RV for the classifications of the 23 referring physicians when referenced to those of the nuclear medicine physicians. The median squared kappa coefficient for all referring physicians was 0.92 (0.90 for general practitioners, 0.93 for internists and 0.92 for cardiologists). The median PA for all referring physicians was 72% (65% for general practitioners, 73% for internists and 71% for cardiologists).

**Table 1:** Analysis of the assessment of ischemia

**Physician**	**Specialty**	**Title**	**K_sq**	**PA**	**RC**	**RP**	**RV**
1	GP	S	0.83	35	0.736	0.251	0.000
2	GP	R	0.93	65	−0.007	0.139	0.007
3	GP	S	0.89	65	−0.192	−0.003	0.016
4	GP	R	0.87	55	0.295	0.030	0.033
5	GP	R	0.95	72	−0.210	0.054	0.005
6	GP	R	0.89	65	0.120	0.090	0.034
7	GP	S	0.91	72	0.085	−0.078	0.015
8	GP	S	0.96	75	−0.283	0.013	0.000
9	IM	R	0.93	73	−0.219	0.038	0.002
10	IM	R	0.96	73	−0.188	0.031	0.004
11	IM	R	0.95	73	−0.149	0.018	0.006
12	IM	R	0.92	72	−0.027	0.113	0.006
13	IM	S	0.92	82	−0.111	−0.067	0.007
14	IM	R	0.87	45	0.374	0.142	0.031
15	IM	S	0.93	65	0.128	0.039	0.016
16	C	S	0.94	72	−0.165	0.040	0.002
17	C	S	0.89	55	0.356	0.108	0.010
18	C	R	0.73	63	0.147	−0.063	0.027
19	C	S	0.95	75	−0.067	0.023	0.005
20	C	S	0.96	80	−0.105	0.004	0.001
21	C	S	0.97	80	−0.149	0.018	0.002
22	C	R	0.90	65	0.103	−0.036	0.010
23	C	S	0.90	70	−0.189	−0.048	0.006
Median			0.92	72	−0.067	0.030	0.006

GP – general practitioner, IM – internal medicine, C – cardiologist, S – specialist, R – resident, K_sq – squared kappa, PA – percentage agreement, RC – relative concentration, RP – relative proportion, RV – relative rank variance.

The results from one physician (general practitioner #1) whose classifications for ischemia were systematically shifted in concentration towards grades 2–4 (central grade categories) are shown in Table 2A. The RC for this physician was highly positive (0.74). The results for another physician (general practitioner #8) whose classifications were systematically shifted in concentration towards grades 1 and 5 are shown in Table 2A. The RC for this physician was negative (−0.28). The results from a physician (cardiologist #4) who had the median value of RC (−0.067) are shown in Table 2C.

**Table 2:** **Classifications for ischemia for three individual referring physicians; general practitioner #1 (A), #8 (B) and cardiologist #4 (C).** 1 = no ischemia, 2 = probably no ischemia, 3 = equivocal, 4 = probable ischemia, 5 = certain ischemia


**A.**							
		**Physicians in nuclear medicine**					
		1	2	3	4	5	Total
GP#1	5						0
	4				14	9	23
	3						0
	2	30	5				35
	1	2					2
	Total	32	5	0	14	9	60
GP – general practitioner							
**B.**							
		**Physicians in nuclear medicine**					
		1	2	3	4	5	Total
GP#8	1				10	19	19
	2				4		4
	3						0
	4						0
	5	32	5				37
	Total	32	5	0	14	9	60
GP – general practitioner							
**C.**							
		**Physicians in nuclear medicine**					
		1	2	3	4	5	Total
C#4	1				6	7	13
	2				7	2	9
	3	1			1		2
	4	2	2				4
	5	29	3				32
	Total	32	5	0	14	9	60
C – cardiologist							

GP – general practitioner, IM – internal medicine, C – cardiologist, S – specialist, R – resident, K_sq – squared kappa, PA – percentage agreement, RC – relative concentration, RP – relative proportion, RV – relative rank variance.

### **Analysis of classification of infarction**

Squared kappa values, PA, RC, RP and RV for all physicians are shown in Table 3. For the 23 referring physicians, the median squared kappa coefficient was 0.92 (0.89 for general practitioners, 0.92 for internists and 0.93 for cardiologists). Median PA was 72% (73% for general practitioners, 72% for internists and 73% for cardiologists).

**Table 3:** Analysis of the assessment of infarction

**Physician**	**Specialty**	**Title**	**K_sq**	**PA**	**RC**	**RP**	**RV**
1	GP	S	0.80	18	0.740	0.173	0.024
2	GP	R	0.88	73	0.029	−0.025	0.007
3	GP	S	0.89	80	−0.057	−0.032	0.010
4	GP	R	0.84	43	0.457	0.031	0.022
5	GP	R	0.96	82	−0.055	−0.016	0.020
6	GP	R	0.88	72	0.180	0.098	0.001
7	GP	S	0.89	60	0.317	−0.058	0.001
8	GP	S	0.96	75	−0.079	−0.023	0.005
9	IM	R	0.93	75	−0.129	0.083	0.007
10	IM	R	0.87	72	−0.158	0.066	0.014
11	IM	R	0.90	75	0.092	0.078	0.004
12	IM	R	0.92	70	0.171	0.079	0.002
13	IM	S	0.92	78	−0.098	−0.088	0.001
14	IM	R	0.92	58	0.288	0.063	0.006
15	IM	S	0.95	70	0.151	0.030	0.004
16	C	S	0.95	80	0.070	0.026	0.004
17	C	S	0.92	62	0.310	0.049	0.000
18	C	R	0.74	70	0.055	−0.035	0.045
19	C	S	0.92	67	−0.049	0.058	0.009
20	C	S	0.96	83	−0.080	−0.021	0.002
21	C	S	0.97	80	−0.107	0.059	0.000
22	C	R	0.85	63	0.075	−0.064	0.019
23	C	S	0.95	77	−0.138	0.001	0.002
Median			0.92	72	0.055	0.030	0.005

GP – general practitioner, IM – internal medicine, C – cardiologist, S – specialist, R – resident, K_sq – squared kappa, PA – percentage agreement, RC – relative concentration, RP – relative proportion, RV – relative rank variance.

### **Analysis of the 17-segment model for ischemia**

Considering all 60 patients and thus the 17*60 = 1020 total segments evaluated, the physicians in nuclear medicine marked a total of 65 segments as ischemic and 955 as non-ischemic. A statistically significant underestimation of the ischemic area (compared to the estimates from the physicians in nuclear medicine) was noted for 12 of the 23 referring physicians, a statistically significant overestimation for none, and no statistically significant differences for the remaining 11 referring physicians.

### **Analysis of the 17-segment model for infarction**

The physicians in nuclear medicine marked a total of 75 segments as infarcted and 945 as non-infarcted. A statistically significant underestimation of the infarcted area (compared to the estimates from the physicians in nuclear medicine) was noted for 6 of the 23 referring physicians, a statistically significant overestimation for one, and no statistically significant differences for the other 16 referring physicians.

### **Equivocal cases**

**Figure 3:**
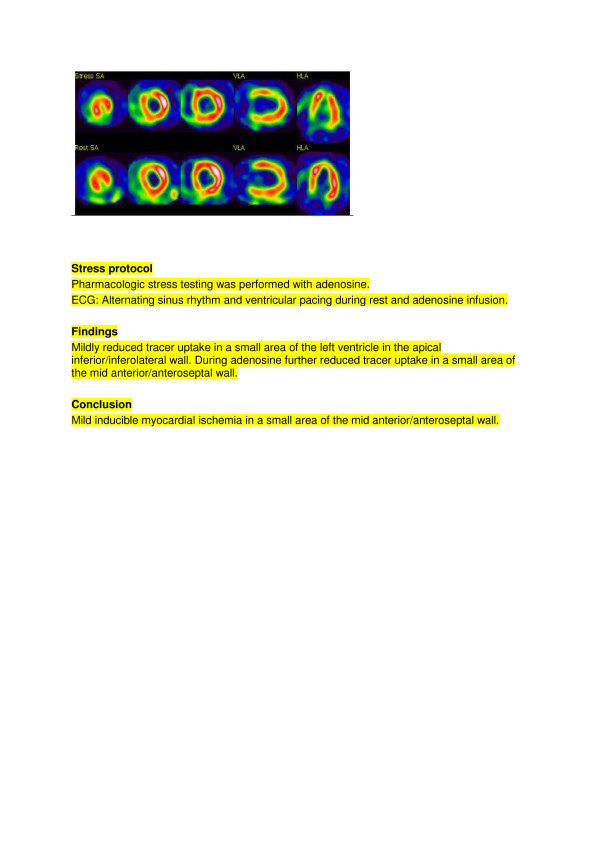
Example of an MPS test result reported as “equivocal” with regard to infarction.

Whereas in no report in this study was ischemia classified as “equivocal” by the physicians in nuclear medicine, the infarction result was ultimately classified as “equivocal” in 3 cases (example shown in Figure 3). Of the referring physicians, only 4, 1 and 0 correctly identified these 3 respective cases as “equivocal”. The remaining physicians varied in their answers from “no infarction” to “certain infarction”. In all 3 cases, the physician in nuclear medicine did not write anything about fixed defects/infarcts in the conclusion section, and only used verbiage such as “inhomogeneous tracer distribution in the … wall at rest” or “mildly reduced tracer distribution…” etc., in the description section of the scintigraphy findings.

## **Discussion**

The principal aim of this study was to investigate whether referring physicians understand the final MPS report as intended by the physician responsible for the image interpretation at the nuclear medicine department. Generally, there was a good agreement between the physicians at the nuclear department interpreting the examination and the referring physicians with a high squared kappa coefficient and a reasonably high PA. In a few cases, the PA was less than 50% for the classifications of both ischemia and infarction. The squared kappa coefficients were high despite these low PAs since the classifications made by the referring physicians were close to the ones made by the physician at the nuclear department on the five-grade scale. The explanation for some of the low PAs was systematic differences in RC, more apparent when evaluating ischemia than infarction. For example, the general practitioner described in Table 2A had a low PA and a high RC, but his classifications did not deviate by more than 1 grade on the five-grade scale from the classifications made by the physicians in nuclear medicine. High absolute RV values were explained by high absolute RC values, since many of the patients did not have ischemia or infarction (n = 32 had no ischemia; n = 31 had no infarction). Thus the differences between physicians were due to systematic differences in RC and not by random differences or differences in RP, and there was no under- nor over classification with respect to the main diagnosis but only with respect to the extent of ischemic/infarcted area. Systematic differences in RC values could possibly be explained by the different practice styles of the referring physicians, i.e., the extent to which a referring physician typically completely “trusts” a diagnostic test report (see Table 2A and Table 2B).

For the classification of infarction, it was clear that when the physician in nuclear medicine was uncertain (equivocal) and did not write anything about the presence or absence of infarction in the conclusion section of the report, the referring physicians did not understand the message. In these cases, there was a large difference in the interpretations made by the referring physicians, and they used the whole range from “no infarction” to “certain infarction” for their interpretations. Thus, it is advisable for the physician at the nuclear medicine department to clearly state the presence versus absence of infarction, or its uncertainty, in the conclusion section of the report.

Different departments of nuclear medicine as well as individual physicians have their own way of writing MPS reports. Traditions in vocabulary could easily be understood within the local hospital, but could be a challenge when exchange of patient data across multiple users or institutions is necessary. In Malmö, where the present study was performed, the EXINI heart^TM^ software package is used. The program not only shows the images, but also interprets the study by using computer assisted diagnosis and writes a preliminary report based on the computerized interpretation. The physician responsible for the interpretation changes the final report according to his/her interpretation. If the interpretation made by the software is considered correct (most often this occurs in patients with normal results), there is no need for the physician to change the phrasing in the final report. Thus, the reports written in Malmö, at least for normal studies, are already fairly standardized. Hospitals not using templates or structured reports might have a larger variety in their phrasing, thus making it even more difficult for referring physicians to understand the message.

Overall, there was a tendency to underestimate the area of ischemia and infarction by the referring physicians. This is potentially troublesome because treatment regimens may differ when ischemia involves only a small versus large area of the heart (e.g., medical treatment is generally preferred if less than 10% of the heart is ischemic whereas percutaneous coronary intervention may be preferred if more than 10% is ischemic) [14]. The physicians at the nuclear medicine department in Malmö use the 17-segment model for describing the locations of ischemic and infarcted areas (using words such as apical, mid-ventricular, basal, anterior, anteroseptal, septal, etc.) instead of giving the total percentage of ischemic/infarcted areas.

In general we would propose the use of concise, clear-cut conclusions (presence or absence of ischemia and infarction) with an extent of the abnormalities expressed as number of involved segments or as a percentage of the left ventricle.

### *Study limitations*

Our study has several potential limitations. For example it is possible that inclusion of images in the final report affected our results, although this is standard procedure at our laboratory and encouraged by current guidelines [10]. Some referring physicians might have classified the report based on the images rather than the phrases used by the physician in nuclear medicine.

None of the 60 cases in the study were classified as “equivocal” with regard to ischemia by the physicians in nuclear medicine. Since inducible ischemia is the most important diagnosis in MPS, it would have been interesting and important to know how an “equivocal” ischemic finding would have been interpreted by the referring physicians.

Another limitation is that we did not ask the study participants to specify the vessel or vessels affected, something that might also have been of interest.

We used a limited number of referring physicians in the study, and used only reports written by physicians in nuclear medicine at the same single hospital. It is possible that the results would have been different had we included more referring physicians, and reports from several different hospitals.

There is also a possibility that the referring physicians who chose to participate in the study were more interested in MPS than the 7 physicians who declined.

## **Conclusions**

Whereas referring physicians demonstrate a good understanding from MPS reports of the general presence versus absence of ischemia or infarction in the referred patient, they often underestimate the extent of any ischemic or infarcted area. This may have adverse clinical consequences and thus the language in final reports from MPS tests might be further improved and standardized.

## Abbreviations

MPS: Myocardial Perfusion Scintigraphy; GP: General Practitioner; IM: Internal Medicine; C: Cardiologist; S: Specialist; R: Resident; K_sq: Squared Kappa; PA: Percentage Agreement; RC: Relative Concentration; RP: Relative Proportion; RV: Relative Rank Variance.

## Competing interests

LE and MO are stockholders of EXINI Diagnostics.

## Authors’ contributions

ET participated in the design of the study, helped with the statistical analysis and drafted the study. PH performed the statistical analysis and helped to draft the manuscript. MO and MW participated in the design of the study and helped to draft the manuscript. LE participated in the design of the study, helped with the statistical analysis and helped to draft the manuscript. All authors read and approved the final manuscript.
